# Exploring the *P. falciparum* antigens associated with reduced risk of malaria in pregnancy

**DOI:** 10.3389/fimmu.2025.1622435

**Published:** 2025-07-14

**Authors:** Lucy Mwai, Sebastian Musundi, Harrison Waweru, Hikaru Nagaoka, Purity Gacheri Limbua, Takafumi Tsuboi, Eizo Takashima, Jesse Gitaka, Bernard N. Kanoi

**Affiliations:** ^1^ Centre for Malaria Elimination, Institute of Tropical Medicine, Mount Kenya University, Thika, Kenya; ^2^ Division of Malaria Research, Proteo-Science Center, Ehime University, Matsuyama, Japan; ^3^ School of Pure and Applied Sciences, Mount Kenya University, Thika, Kenya; ^4^ Division of Cell-Free Sciences, Proteo-Science Center, Ehime University, Matsuyama, Japan

**Keywords:** malaria in pregnancy, placental malaria. gravida, antibodies, immunity, bloodstage proteins

## Abstract

*Plasmodium falciparum* infection in pregnancy leads to substantial maternal and infant morbidity and mortality. Such an infection may result in placental malaria (PM) due to *P. falciparum*-infected red blood cells adhering to the placenta via parasite-derived ligands. Despite the risk of infection being the same for women of all gravidities, the risk of poor birth outcomes is highest in primigravida women as they lack protective antibodies against placental malaria-associated parasites. Thus, understanding how specific *P. falciparum* antigens interact with the host’s immune system during the first and subsequent pregnancies may provide insights into the immunopathology of malaria and guide vaccine target prioritization. In this study, we assessed human antibody responses to 698 P*. falciparum* recombinant antigens derived from different antigen families among Kenyan primigravida and multigravida women. Consistent with existing literature, we observed high immunoreactivity across the different antigen families, with the number of antigens identified by sera from pregnant women increasing with gravidity. Antibody response analysis selected 3 antigens: PF3D7_1301800 (SURFIN 13.1), PF3D7_0424400 (SURFIN 4.2), and PF3D7_1252100 (rhoptry neck antigen 3 domain) as statistically significant in multigravida. While all five VAR2CSA domains were immunoreactive with seroprevalence of 42 - 62% and correlated with the selected antigens, which suggests co-acquisition, none had statistical significance in association with gravidity. Thus, although further characterization of the selected antigens will be required, this study may provide insights into targets that could be prioritized for vaccine development to reduce risks associated with malaria in pregnancy.

## Introduction

In regions of malaria endemicity, pregnant women are at high risk of *Plasmodium falciparum* infection, which could lead to infection of the placenta leading to placental malaria (PM) (1). PM accounts for about 10,000 maternal and 200,000 infant deaths worldwide, mostly due to inflammatory and adverse outcomes, namely severe maternal anemia and low birth weight (2). With over 25 million pregnancies at risk of infection in sub-Saharan Africa and loss of efficacy of sulfadoxine-pyrimethamine intermittent preventive treatment during pregnancy due to the emergence of *P. falciparum* resistance to the drug (3), it calls for the development of new interventions. Malaria vaccines offer the best approach for reduced transmission in addition to other tools currently available for malaria control, including vector control, chemoprophylaxis, prompt diagnosis, and use of effective anti-malarial drugs (4).

Association between levels of antibodies and the risk of clinical malaria has pointed to an important role of *P. falciparum* antigens in children and malaria in pregnancy (MiP) (5–7). However, the effector mechanisms of these antibodies are incompletely understood. In pregnancy, multigravida mothers are at lower risk of PM-associated complications mainly due to the acquisition of protective antibodies that are thought to prevent the accumulation of infected red blood cells (iRBCs) in the intervillous space of the placenta (8). The majority of studies, including vaccine trials, focus on understanding and evaluating the role played by VAR2CSA, a unique member of *P. falciparum* erythrocyte membrane protein 1 (PfEMP1) family that is upregulated in placental malaria-associated parasites, that has been associated with adverse pregnancy outcomes by its interaction with placental chondroitin sulphate A (CSA) (9). It was recently demonstrated that Cameroonian women who were negative for PM at delivery had significantly higher antibody levels to the Full-length VAR2CSA (FV2) region of VAR2CSA throughout the pregnancy and that women with a high proportion of high avidity antibody to the FV2 region during the second trimester had a reduced risk of having PM at delivery (10). Indeed, VAR2CSA is the only antigen under consideration as a malaria vaccine for malaria in pregnancy (11–14). However, recent studies have identified some invariant red blood cell plasmodium surface proteins co-expressed with VAR2CSA such as; PF3D7_0424000 and PF3D7_0936900, which are Poly-Helical Interspersed Sub-Telomeric (PHIST) exported proteins, PF3D7_0202400 a Plasmodium Translation Enhancing Factor (PTEF) and the *P. falciparum* chondroitin sulfate A ligand, (PfCSA-L; PF3D7_1001000) whose mechanism in PM pathogenesis could be explored (15–17). There is inconsistent data regarding the molecules to target for vaccine development, highlighting the urgent need for in-depth studies to identify the ideal proteins of *P. falciparum* that can generate functionally protective antibodies against MiP. This study aims to identify these target proteins of *P. falciparum* in a region of stable malaria transmission in Kenya, with the goal of exploring their potential in the development of placental malaria vaccines.

## Materials and methods

### Study population

The serum samples used in this study were obtained from a well-characterized biobank of a prospective cohort of pregnant Kenyan women (n=53) between 12–18 weeks of gestation visiting Webuye County Hospital, Bungoma County, Kenya. The county is located in a malaria hyperendemic region where residents have been exposed to malaria infection since childhood (18, 19). The study included women who were under routine intermittent preventive treatment in pregnancy (IPTp) with sulfadoxine-pyrimethamine (IPTp-SP), and who were in the first or early second trimester of pregnancy. Serum samples were collected 4 weeks after the IPTp, immediately frozen, and shipped on dry ice to the laboratories at Mount Kenya University, where they were carefully stored uninterrupted in a -80°C deep freezer. Each serum sample was linked to its corresponding anonymized demographic data and pregnancy outcome information gathered during both scheduled and unscheduled hospital visits. The study excluded women with conditions such as tuberculosis or other known comorbidities.

### Production of a *P. falciparum* parasite antigen library

The assayed antigens, produced using the wheat germ cell-free system (WGCFS), consisted of broad range of asexual blood-stage proteins (BSP; n = 158) and variable surface antigens (VSAs);*P. falciparum* erythrocyte membrane protein 1 (PfEMP1): Duffy binding–like domains (DBL; n= 163) and cysteine-rich interdomain regions (CIDR; n = 108), repetitive interspersed family proteins (RIFINs; n =182), surface-associated interspersed gene family proteins (SURFINs; n = 33), and subtelomeric variable open reading frame proteins (STEVORs; n= 54) as previously reported (20–22). VSAs are predominantly expressed on the surface of infected erythrocytes during the trophozoite-schizont stages. These 698 *Plasmodium falciparum* antigens included in this study were prioritized based on their previously reported serological reactivity, functional relevance in parasite-host interactions (e.g., cytoadherence, immune evasion), and expression during the asexual blood stage—the primary stage associated with clinical disease.

Briefly, the transcription templates for the 698 proteins were prepared from genomic DNA or complementary DNA of *P. falciparum* 3D7 strain amplified by polymerase chain reaction using high fidelity PrimeSTAR DNA polymerase (Takara Bio, Kusatsu, JP), and cloned into pEU plasmid vector (CellFree Sciences, Matsuyama, Japan) with In-Fusion HD Cloning Kit. The N-terminus His-tagged mono-biotinylated recombinant proteins were expressed by the WGCFS (21). Protein expression was confirmed by Western blot analysis using HRP-labelled streptoavidin (21).

### Antibody quantification by AlphaScreen assay

To assess the level of acquisition of anti-*P. falciparum*-specific antibodies in the malaria-exposed pregnant women cohort, we performed an AlphaScreen assay (PerkinElmer) with all the 698 recombinant proteins as described (23). The AlphaScreen assay readout was presented as AlphaScreen Counts (ASC). To standardize assay variability, serially diluted biotinylated rabbit IgG (PerkinElmer) was included in each plate. The assays were run in randomized order.

### Statistical analysis

The data analysis was performed using the R software (Version 4.2.1, R Foundation for Statistical Computing). The antigen seropositivity cut-off value to human sera was set above the assay background readout, and an antigen was considered immunoreactive if more than 10% of the participants had ASC levels above the seropositivity cut-off. A linear regression model was used to evaluate significant associations of antibody breadth based on age and hemoglobin levels. Kruskal–Wallis test with pairwise adjustments was used to evaluate the association of antibody breadth between primigravida and multigravida women groups. Then, a volcano scatter plot was generated; which is a graphical representation of a differential antibody response analysis to evaluate and represent changes in antibody response to *P. falciparum* antigens between primigravida and multigravida. P < 0.05 was considered significant.

## Results

### Characteristics of the pregnant women in this study

The serum samples used in this prospective study were obtained from a cohort of pregnant women visiting Webuye Level IV Hospital, Bungoma County, Kenya (n = 53). The participants were spread across gravida 1 to 5 and were aged between 19–42 years. Their ages were significantly different across gravida groups (Kruskal Wallis test, p < 0.05) (**Table 1**). The overall mean hemoglobin level was 13.3 g/dl. There were four *P. falciparum*-positive malarial infections at enrollment by rapid diagnostic test (RDT). The majority of the births had normal newborn birth weight, with a mean of 3.22kg. Only one miscarriage was observed.

**Table 1:** Baseline characteristics of the pregnant women study population.

Characteristics	Gravida stratification					Overall	p-value ~
	1	2	3	4	5		
Number of participants [n]	12	17	13	6	5	53	
Age [Years] `	25 [19 – 36]	26 [20 – 35]	28 [20 – 37]	26 [21 – 36]	37 [33 – 42]	27 [19 – 42]	0.001
HB [g/dl] #	13.75 [1.75]	13.2 [1.54]	13.54 [1.59]	14.15 [1.88]	11.36 [1.07]	13.34 [1.70]	0.051
Immunoreactive Antigens #	410 [113.86]	415.18 [143.70]	446.38 [139.88]	356.33 [81.88]	282.8 [169.45]	402.51 [136.51]	0.194
Malaria infection at enrollment (by RDT)	1 [8.3]	1 [5.9]	1 [7.7]	0 [0.0]	1 [20.0]	4 [7.5]	0.794
Pregnancy Outcome Alive *	12 [100.0]	16 [94.1]	13 [100.0]	6 [100.0]	5 [100.0]	52 [98.1]	0.707
Newborn Birth Weight [Kgs] #	3.24 [0.28]	3.23 [0.25]	3.1 [0.32]	3.48 [0.22]	3.24 [0.36]	3.22 [0.29]	0.185

^¶^Median and [Interquartile range].

*Number [Percentage in each group].

^#^Mean [Standard Deviation].

∼Kruskal-Wallis rank sum test, Significance <0.05.

### Seroprevalence of serum antibodies to various *P. falciparum* antigens

**Figure 1:**
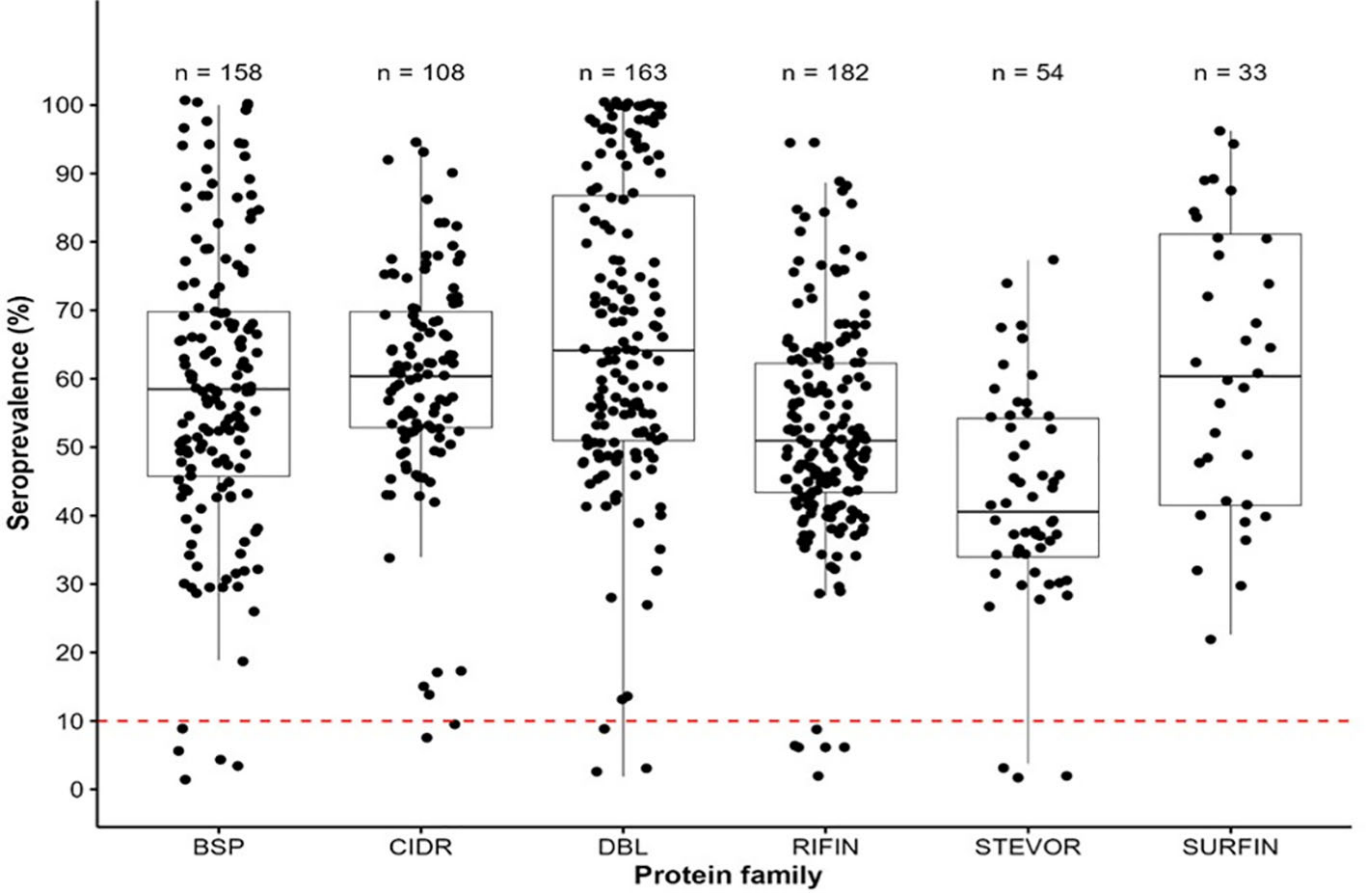
Immunoreactivity and seroprevalence of antibodies to *Pf* antigen families (BSPs, CIDRs, DBLs, RIFINs, STEVORs, and SURFINs). (Antibody immunoreactivity in the cohort of pregnant women to asexual blood stage antigens (BSP), CIDR (cysteine-rich interdomain regions of PfEMP1), DBL (Duffy binding–like domains of PfEMP1), RIFIN (repetitive interspersed family proteins), SURFIN (surface-associated interspersed gene family proteins) and STEVOR (subtelomeric variable open reading frame proteins). Box plots illustrate the overall medians of each protein group at the horizontal line per group. The dashed red horizontal line indicates a 10% seroprevalence that was set as the antigens immunoreactivity cut-off point.

To determine the seroprevalence among the pregnant women in the cohort, we evaluated the reactivities of the serum obtained from the pregnant women (n=53) against a library of recombinant antigens (n=698) (**Supplementary Table S1**). The seroprevalence variedsignificantly among the antigen groups: BSP (3.8-100%), CIDR domains (7.6-94.3%), DBL domains (1.9-100%), RIFIN (1.9-94.3%), STEVORs (1.9-77.4%) and SURFINs (22.6-96.2%) (**Figure 1**). The DBLs had the highest seroprevalence, with a median of 64.2%, while STEVORs had the lowest median of 40.6%. All the SURFINs were immunoreactive with a seroprevalence above the threshold cut-off point of 10%. Only a small number of BSP, CIDR, DBL, RIFIN, and STEVOR were not immunoreactive (Kruskal-Wallis rank; P< 0.05). The observed antibodies against the *P. falciparum* library indicated that this population was previously exposed to malaria infection. Therefore, the observed outcomes were influenced by the abundance or efficacy of this immunity.

### Relationship between antibody breadth with age, hemoglobin levels, gravida, and malaria infection status

**Figure 2:**
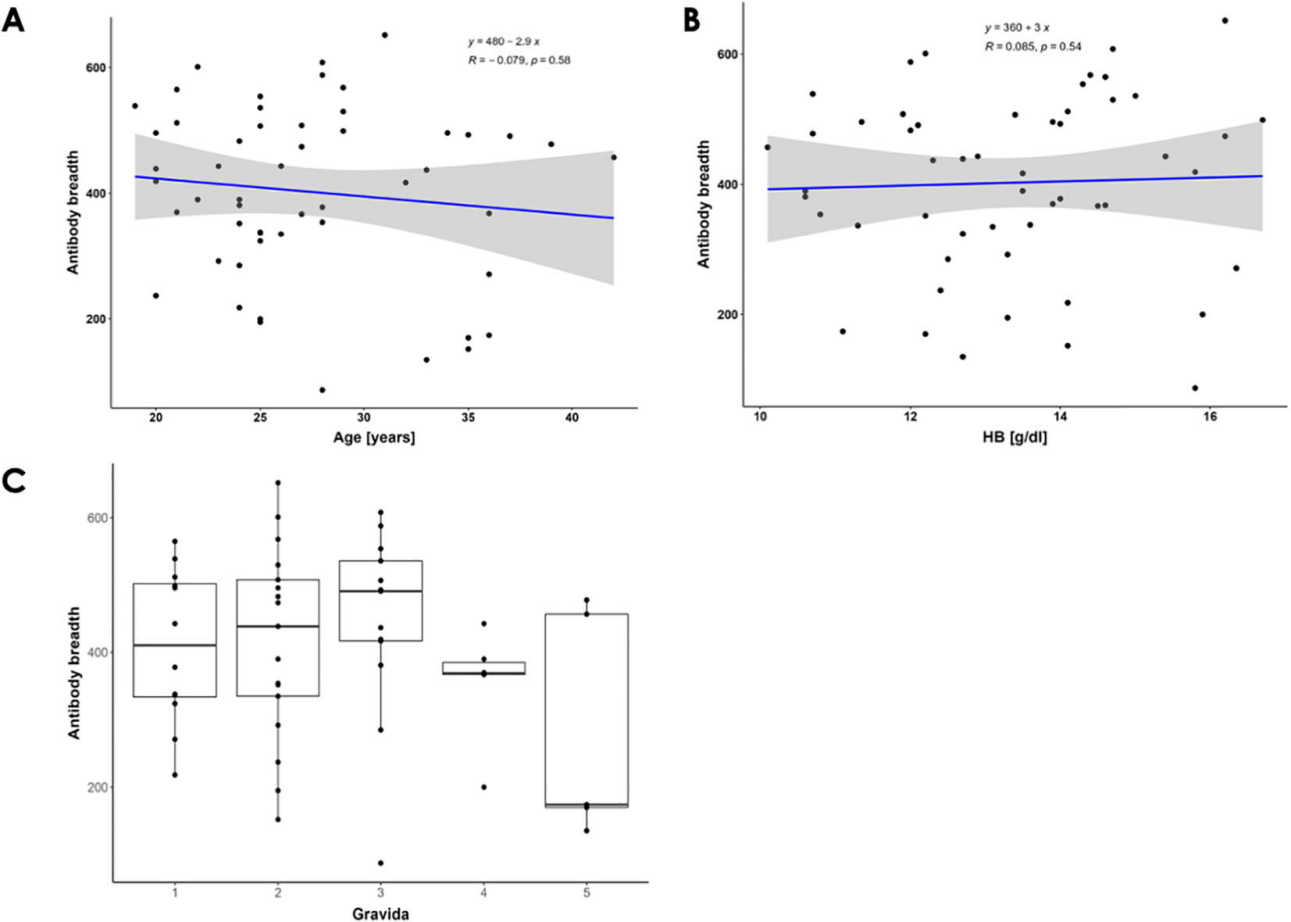
Breadth of Antibody Responses Against P. falciparum Antigens. **(A)** Correlation between the breadth of antibody responses and pregnant women's ages. Spearman's correlation coefficients (R) and significance P-values are shown. The blue line represents the linear regression line, while the gray area indicates the 95% confidence interval. There is a non-significant negative correlation between the age of participants and the number of P. falciparum antigens recognized. **(B)** Correlation between the breadth of antibody responses and hemoglobin levels. The blue line represents the linear regression line. There is a weak, non-significant positive correlation between participants' serum antibodies and hemoglobin levels. **(C)** Distribution of breadth of antibody responses across gravidae 1 to 5. The box plots illustrate the overall medians of antigens recognized (indicated by the horizontal line) per gravida. The medians are: gravida 1 = 410.5, gravida 2 = 439, gravida 3 = 491, gravida 4 = 369, and gravida 5 = 174.

To determine the relationship between the antibody breadth (number of antigens recognized) by age, hemoglobin levels, gravida, and malaria infection status for each participant in the prospective study, we carried out association analysis. The overall median and range of antigens recognized by the participants were 419 and 87- 652, respectively. Further, we generated a linear regression model for the antibody breadth versus the age of participants (**Figure 2A**). No significant correlation was found between antibody breadth and participants’ age (Spearman’s correlation, R = -0.079, P=0.58).

Additionally, PM is characterized by reduced plasma hemoglobin levels due to hemolysis of infected and uninfected red blood cells (24). In this study, the pregnant women cohort showed normal hemoglobin levels above 10 g/dl. We generated a linear regression model for the antibody breadth versus the hemoglobin levels of the participants (**Figure 2B**). No significant correlation was found (Spearman R = 0.085, P = 0.54) between participants’ serum antibodies and hemoglobin levels.

Finally, since the risk of malaria-associated poor birth outcomes decreases with increasing gravidity, making gravida a clear indicator of PM immunity, we assessed the antibody responses in women of different gravida (25, 26). We found that the number of *P. falciparum* antigens recognized by participants’ antibodies increased with the participants’ gravidity up to gravida 3 (**Figure 2C**) (8). This suggests that specific multiple antigens may play a role in protective immunity against PM. We further evaluated the effect of malaria status in multigravida women. Only 3 women had positive malaria infection at enrollment. Positive malaria cases showed a trend of increased antibody response across all protein families except in the STEVOR family, where the median for both positive and negative cases was equal (**Supplementary Figure S1**) (26).

### Differential antibody response analysis by gravida

**Figure 3:**
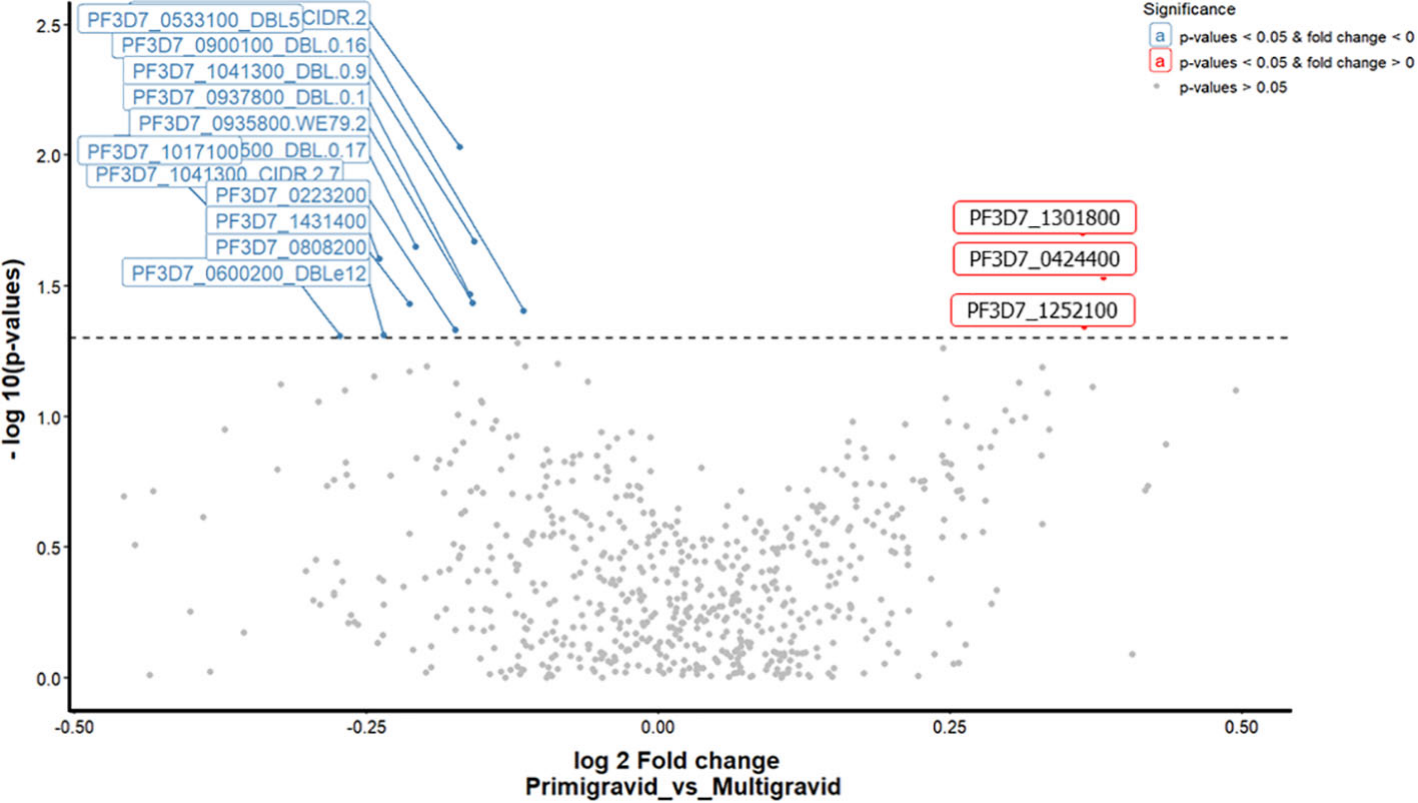
A volcano scatter plot showing differential antibody response analysis between primigravida vs multigravida. This graph indicates the fold change on the x-axis and p-values on the y-axis. The points represent individual mean ASC counts of antigen domains colored according to significance by gravida (Significant values p<0.05, fold change < 0 (blue colour), p<0.05, fold change >0 (red colour), and non - significant values > 0.05 grey colour). The total significant antigens by fdr unadjusted analysis are (13 primigravida and 3 multigravida).

In order to identify which parasite antigens may be responsible for the variation in antibody response between pregnant women, we created a volcano scatter plot to analyze the differential antibody response. This analysis revealed differences in the antibody response to *P. falciparum* antigens between primigravida women (gravida 1) and multigravida women (gravida 2 and 3). We did not include data from women (gravida 4 and 5) due to the limited number of samples available. The mean antibody responses to three of the 698 were significantly higher in multigravida women than in primigravida women (**Figure 3**; **Supplementary Table S2**). Of the three antigen domains, two were SURFIN domains; (PF3D7_1301800 - SURFIN 13.1 and PF3D7_0424400 - SURFIN 4.2, and one was a blood stage protein; (PF3D7_1252100 - rhoptry neck protein 3). However, primigravida women responded significantly to a greater number of *P. falciparum* proteins compared to the multigravida women. Although, two domains of VAR2CSA namely; PF3D7_1200600_CIDRpam and PF3D7_1200600_DBLe10 were recognized by in multigravida women, no significant antibody responses to this antigen was observed.

## Discussion

In malaria-endemic regions, there is a strong negative association between gravidity and poor birth outcomes from placental malaria (PM) (26) such that, poor birth outcomes are mainly experienced by primigravida women. Although there is evidence showing that the VAR2CA mediates the adhesion of *P. falciparum* parasite to CSA in placenta and that the acquisition of antibodies against this VAR2CSA are parity-dependent, direct epidemiological proof that VAR2CSA antibodies prevent PM and associated adverse pregnancy and birth outcomes has been inconsistent (27). Therefore, this study aimed to investigate antibody responses in a cohort of pregnant women and found a high seroprevalence against antigens expressed in various stages of the malaria parasite’s life cycle, specifically those expressed in the merozoite stage (BSP), and on the surface of iRBCs (PfEMP1, RIFIN, SURFIN, and STEVOR).

Consistent with previous studies, women with successive pregnancies had a wider breadth of antibody response to a broad range of parasite proteins with an increase in gravidity, for gravida 1 to 3 (26–29). The analysis of the breadth of antibody response to the antigen library was further extrapolated based on age, hemoglobin levels and the differential antibody response based on gravida (**Figure 3**). Through these analyses, we explored the key antigenic targets that are attributed to the observed differences in gravidity namely; PF3D7_1301800 (SURFIN 13.1) and PF3D7_0424400 (SURFIN 4.2), and PF3D7_1252100 (rhoptry neck protein 3, PfRON3). These antigens had seropositivity of 30%, 62%, and 69%, respectively, in the pregnant women cohort. SURFIN family antigens have been reported as targets of naturally acquired immunity against malaria infections (21). The PF3D7_1301800 (SURFIN 13.1) is understudied but has been predicted to be involved in cell surface adhesion, hence supporting the sequestration of iRBCs (30). On the other hand, PF3D7_0424400 (SURFIN 4.2) has been established to colocalize with PfEMP1 on the iRBCs membrane as well as the merozoite surface, hence it could also be important for parasite growth in the blood-stage development (29, 30). PfRON3 (blood stage antigen) has been shown to form a complex with *P. falciparum* rhoptry-associated membrane antigen (PfRAMA), resulting in a novel PfRON3/PfRAMA rhoptry antigen complex on the *P. falciparum* merozoite. The complex has a role in the invasion of red blood cells by merozoites (31, 32).

In most studies on placental malaria, VAR2CSA—a member of the PfEMP1 family—has been intensively investigated as a vaccine candidate using laboratory parasite strains (10, 33). In contrast, our study did not identify VAR2CSA domains among the key antigens associated with gravidity, although PF3D7_1200600_DBLe10 and PF3D7_1200600_DBLpam3 were immunoreactive in 60% and 45% of participants, respectively. Such discrepancy with prior findings may be attributed to differences in the study design employed, notably the timing of antibody measurement, since VAR2CSA‐specific IgG may peak later in pregnancy or postpartum and be missed in cross-sectional assays like the one used in this study (26, 34). Another factor could be population genetics, given that VAR2CSA exhibits extensive allelic diversity and gene duplication in African field isolates this may have lead to potential underestimation of antibody responses when using laboratory strain–derived antigens in prior studies (35, 36). Methodological differences, such as the use of truncated versus full-length VAR2CSA constructs, assay platforms, and antigen folding can further influence epitope presentation and detection sensitivity (37, 38).

Our findings therefore challenge the universality of the current VAR2CSA-focused vaccine strategies, that is, PRIMVAC and PAMVAC, which aim to elicit inhibitory IgG against specific CSA-binding domains (39, 40). To broaden protective coverage across diverse field isolates, inclusion of multiple VAR2CSA variants or epitopes and supplementary antigens such as SURFIN 13.1, SURFIN 4.2 and PfRON3 recognized in this study may be warranted. Ultimately, multicomponent vaccine formulations that combine VAR2CSA domains with other blood-stage or surface antigens hold promise for achieving parity-independent protection against placental malaria (39).

On the other hand, primigravida women participants in this study responded significantly to a greater number of *P. falciparum* proteins. Previous studies have shown that primigravida women are more susceptible to malaria infection during pregnancy (41) because of factors such as pregnancy-related immunosuppression (42) and more recently, the fact that the placenta provides a unique environment for malaria parasite sequestration inducing increased antibody response in women who get pregnant for the first time (43). This placental sequestration of *P. falciparum* is mediated primarily by variant surface antigens on iRBCs, most notably PfEMP1; a family encoded by the var gene repertoire of approximately 50–60 distinct genes per haploid genome (43, 44). Expression of var genes is strictly mutually exclusive, where, only a single PfEMP1 variant is displayed at any given time, through heterochromatin silencing and locus repositioning mechanisms (43, 45),. Periodic switching among var genes drives antigenic variation, allowing the parasite to evade host antibodies and maintain chronic malarial infection (43). In our cohort of primigravida women, seven of the thirteen differentially recognized antigens were PfEMP1 variants, underscoring the extensive heterogeneity of var expression among field isolates and its influence on parity‐dependent immune responses time (46). The most significant seropositive antigen among the PfEMP1 recognized in primigravida women was PF3D7_0533100 (*var1csa*), which was annotated as a pseudogene in the 3D7 strain. However, interestingly, Cabral et al. (44) reported that its expression was not involved in the allelic mutual exclusion of *var* gene transcription, which may explain its highly significant expression in the majority of women in this study.

In summary, this study evaluated a library of 698 P*. falciparum* antigens alongside an extensive analysis of clinical data encompassing various outcomes, such as gravidity, maternal anemia, and birth weight. However, due to the study’s limited sample size, identifying protective associations may have been challenging, particularly among women with a higher gravidity. Nevertheless, the study provides evidence that pregnant women in malaria-endemic areas have significant levels of antibodies against both merozoite and iRBC surface antigens. These antibodies could potentially reduce the risk of adverse outcomes associated with placental malaria, such as malaria-associated anemia and low birth weight, and contribute to overall normal pregnancy outcomes. Further research is needed to validate the identified antigenic targets using a larger sample size. It is also crucial to investigate their role in the opsonization of parasitized red blood cells for phagocytosis, opsonization of merozoites, and complement fixation.

## Data Availability

The raw data supporting the conclusions of this article will be made available by the authors, without undue reservation.

## Supplementary material

Supplementary Figure 1 A box plot showing the malaria infection status versus the median antibody responses across the protein families in multigravida women. Positive malaria cases showed increased antibody response across all protein families except in STEVOR family where the median for both positive and negative cases was equal.
