# Supplementary figures and images for: Exploring the P. falciparum antigens associated with reduced risk of malaria in pregnancy
Source: Front Immunol. 2025 Jul 14;16:1622435. doi: 10.3389/fimmu.2025.1622435 (PMC12301398; doi:10.3389/fimmu.2025.1622435)

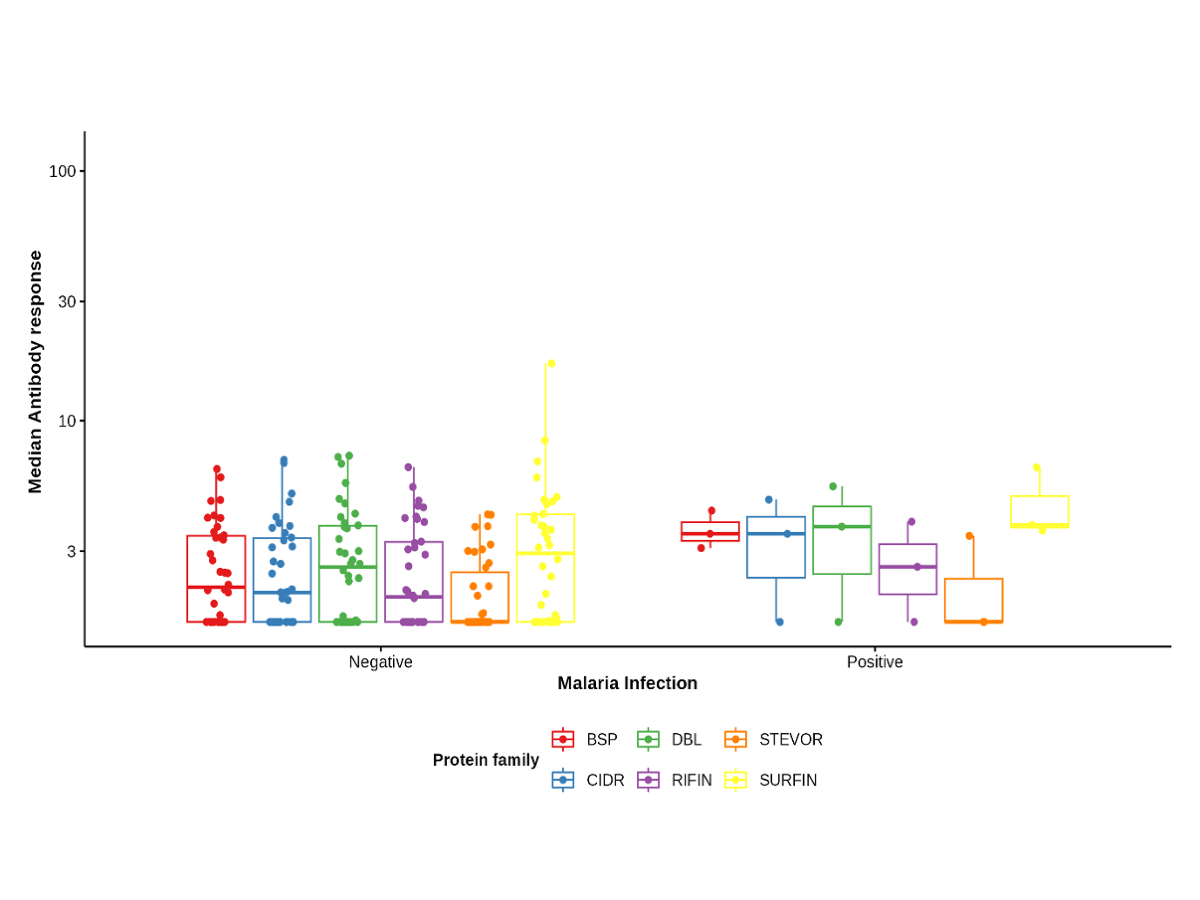

Supplement: Supplementary Figure 1 — A box plot showing the malaria infection status versus the median antibody responses across the protein families in multigravida women. Positive malaria cases showed increased antibody response across all protein families except in STEVOR family where the median for both positive and negative cases was equal. [file Image1.tiff]
